# Magic Diamond: Covalent Bond Formation of Melamine and Other Amines on Nanodiamond Surfaces

**DOI:** 10.1021/acsomega.5c13652

**Published:** 2026-04-17

**Authors:** Tsz Ching Cheung, Camron X. Stokes, Jorge A. Lopez-Rosas, Grace Olivia Drew, Joy Lillian Drew, Anoushka Lakshmi, Nivita Susendran, Muhammed Qasim, Cynthia Melendrez, Sang-Jun Lee, Avery Green, Jia Lu, Virginia Altoe, Dennis Nordlund, Kent Irwin, Abraham Wolcott

**Affiliations:** † Department of Chemistry, 7161 San José State University, 1 Washington Square, San José, California 95192, United States; ‡ Department of Physics, 14770 CUNY-The City College of New York, 160 Covent Ave., New York, New York 10031, United States; § Stanford Synchrotron Radiation Lightsource, 17220 SLAC National Accelerator Laboratory, 2575 Sand Hill Road, Menlo Park, California 94025, United States; ∥ Linac Coherent Light Source, 497525 SLAC National Accelerator Laboratory, 2575 Sand Hill Road, Menlo Park, California 94025, United States; ⊥ Department of Physics, 6429 Stanford University, 450 Jane Stanford Way, Palo Alto, California 94025, United States; # The Molecular Foundry, 1666 Lawrence Berkeley National Laboratory, 1 Cyclotron Road, Berkeley, California 94720, United States; ∇ 588065 Covalent Metrology, 927 Thompson Pl, Sunnyvale, California 94085, United States; ○ 338416 EAG Laboratories, 810 Kifer Rd, Sunnyvale, California 94086, United States

## Abstract

High-temperature, high-pressure (HPHT) nanodiamond (ND) hosts nitrogen-vacancy (NV) centers, solid-state qubits that enable room-temperature quantum sensing by all-optical magnetometry, electrometry, and thermometry. However, the covalent surface functionalization of nanoscale diamond remains largely limited to carboxylate-based chemistries. Amine termination is particularly attractive because theoretical studies predict suppression of midgap states and extended electron-spin coherence times. Recently, chemical activation of alcohol-terminated NDs to alkyl bromides (ND-Br) using SOBr_2_ has enabled nucleophilic substitution through a carbocation intermediate, allowing formation of simple amine terminations. Here, we evaluate whether sterically demanding amines can form covalent diamond–nitrogen bonds on ND-Br surfaces. ND-Br was reacted with branched, linear, and cyclic amines, including polyethylenimine, diethylenetriamine, and melamine. X-ray spectroscopies were used to confirm successful and to probe the resulting electronic structure at the diamond–amine interface. These results expand the chemical toolbox for tuning diamond surface dipoles and electron affinity, providing new pathways for engineering nanodiamond surfaces for quantum sensing and photocatalysis applications.

## Graphical abstract

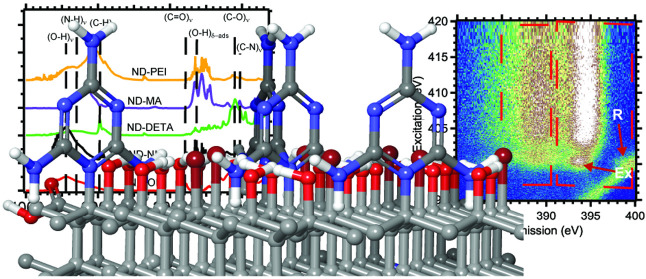

## Introduction

Chemical modification of diamond surfaces can be exploited in several ways to generate new covalent bonds for surface dipole engineering, to remove midgap states for quantum sensing, and to enable photocatalytic chemical transformations. Emergent technologies using nitrogen vacancy (NV) centers and other color centers in diamond have been demonstrated in photonics,^1^ biosensing,^2–4^ quantum metrology,^5–7^ quantum communication,^5,8–10^ electron–hole transport,^11^ and memory storage.^12^ In a fashion similar to nuclear magnetic resonance spectroscopy, the NV center allows for optically detected magnetic resonance or ODMR, yet the magnetometry occurs at room temperature and in customizable setups. While near-surface NV centers have been explored extensively, there are still major questions concerning the identity and removal of electronic noise sources that negatively impact spin coherence times.^13^ Motivation for amination chemistry on diamond in our work is driven by NV-based sensing and photoemission from diamond surfaces for chemical transformations.

Amination or addition of amine groups to diamond surfaces was shown to reduce midgap electronic states using density functional theory (DFT) calculations.^14^ Chou et al. motivated their work to increase the electron-spin coherence properties of NV centers by removing trap states within the NV^–^ center electronic structure. Experimentally, the effect of amine chemistry on near-surface NVs hosted in chemical vapor deposition (CVD) diamond was tested via plasma treatment in under 1 min, and their NV photophysics were characterized.^15^ Abendroth and co-workers reported mixed findings including (1) a reduction in NV fluorescence post-amination, (2) subtle improvements in pulsed-ODMR contrast, and (3) an improvement in the T_2,CPMG_ of selected single NVs. Amination via plasma treatment, while convenient, causes surface roughening due to the high-energy impact of the technique; this issue is especially troublesome for NV-related sensing protocols as highlighted previously.^13^ More recently, Rodgers, Knowles, and De Leon presented diamond surface chemistry with hydrogen-terminated (100) diamond and performed chemical transformations using photoinitiated radical chemistry, including the addition of amines and amides.^16^ Generally, we speculate that mild wet chemical routes may prove to be the most effective at reducing surface roughening while maintaining electron-spin coherence times.

In work related to photoemission into solutions for chemical transformations, amination of electrochemical grade diamond (Element 6) was accomplished with ammonia (NH_3_) plasma under low power conditions of 25 W and high pressures (1.6 Torr), resulting in high yields over 10 min.^17^ Amine termination allowed for the photocatalytic reduction of H_2_ → NH_3_ in a diamond-water cell similar to hydrogen termination, whereby photoemitted electrons are solvated in aqueous conditions with reductive potential.^18^ Surprisingly, electron injection into water by oxygen-terminated diamond hosting NV centers and P1 centers (substitutional nitrogen) has also been observed using 532 nm light and reveals that much is yet to be understood about diamond-water interfaces.^19^ To better understand charge transfer processes from diamond and surface defects, Chemin and Petit used a myriad of overlapping techniques that probe diamond–vacuum, diamond–N_2_, diamond–air, and diamond–electrolyte interfaces and should be informative to researchers in both the charge transfer and quantum sensing fields.^20^ There is speculation that emission of electrons at the diamond-water interface may influence ^1^H sensing when water is confined within nanochannels, and the diffusion rates of water are greatly reduced.^21,22^

Diamond, while inert, can be chemically activated using a dense assembly of good leaving groups as a prerequisite. Discussions of surface termination and surface energy should always consider the two dangling bonds of the (100) surface and the one dangling bond of the (111) surface of CVD or high-pressure high-temperature (HPHT) diamond, respectively. To this end, halogenation of bulk and nanoscale diamond surfaces provides labile leaving groups, enabling the formation of reactive surface intermediates for nucleophilic addition. Treatment of HPHT nanodiamond (ND) with SOBr_2_ efficiently converts alcohol-rich surfaces into brominated intermediates suitable for amine chemistry, with approximately 50% conversion at room temperature.^23^ Density functional theory (DFT) predicted a carbocation-like species on the diamond surface after bromide desorption, one that had sp^2^ character similar to molecular carbocation intermediates and would be susceptible to nucleophilic addition.^24^ Bromination has been an outlier (only 2 confirmed studies), while fluorination and chlorination routes are ubiquitous and predicted to achieve 100% surface coverage. Though bromination chemistry has not been widely used for diamond surface chemistry, it serves as a new route to activate the diamond surface without the need for catalysts and mild nucleophiles can be used. Previous bromination strategies of carbon-based nanomaterials are provided in the SI.

Here, we leverage a metastable alkyl-bromide-terminated ND (ND-Br) and probe if larger amine molecules can access the sterically hindered diamond surface. All chemistry was in inert conditions due to the reactivity of ND-Br and produced successful reactions with diethylenetriamine (DETA), polyethylenimine (PEI), melamine (MA), and a NH_3_ control. DETA is a small linear molecule, PEI is a branched polymer of 800 g/mol, and MA is a conjugated nitrogen-rich triazine. A combination of vibrational spectroscopy, laboratory, and synchrotron-based X-ray measurements was collected to confirm diamond surface functionalization and the diamond electronic structure. Diffuse reflectance Fourier infrared transmission spectroscopy (DRIFTS) shows the conversion of alcohol-rich (ND–OH) to amine-terminated NDs, showing modification of hydrophilicity, and with MA termination revealed that close packing and hydrogen bonding between MA molecules is occurring. XPS confirmed that bromine was left at unity conversion rates and discovered that reactivity increased from PEI to DETA to MA. We explain the order of reactivity based on nucleophilicity and steric considerations, with MA’s size and electron donation to lone pairs aiding diamond-nitrogen bond formation. X-ray absorption (XAS) and resonant inelastic X-ray scattering results provided unique C 1s and N 1s spectra, whereby some samples showed new midgap electronic states. Researchers in the fields of biolabeling with NV NDs, quantum sensing applications, and photoemission from diamond surfaces into aqueous solutions should leverage the work as a route to modify the surface dipole moment, charge density and increase linking strategies for covalent bond formation.

## Materials and Procedures

### Materials

High-pressure, high-temperature nanodiamond powders (monocrystalline diamond powder, MSY 0–0.03 μm and MSY 0–0.05 μm) were purchased from Microdiamant, USA. Anhydrous dichloromethane (99.8% #270997), anhydrous pyridine (99.8% #270970), thionyl bromide (97% #251259), ammonia in tetrahydrofuran (0.4 M in THF #718939), propargylamine (98% #P50900), diethylenetriamine (99% D93856), melamine (99% M2659), and branched polyethylenimine (*M_w_* ∼ 25, 000 g/mol by LS, 99% purity, #408727) were purchased from Sigma-Aldrich (St. Louis, MO). Anhydrous dichloromethane (99.7%+ #41835) and anhydrous dimethyl sulfoxide (99.8% #43998) were purchased from Alfa Aesar (Haverhill, MA). Silicon wafers (4 in.) coated with a 10 nm titanium adhesion layer and 100 nm layer of gold were purchased from LGA Thin Films, Inc. (Santa Clara, CA).

### Procedures

#### Alcohol-Rich HPHT Nanodiamond (ND–OH) Preparation and Storage

Oxidation and purification of 30 and 50 nm HPHT nanodiamond (NDs) occurred in a three-zone tube furnace (Thermo Scientific STF55346COMC-1). Typically, 500 mg of NDs were aerobically oxidized in a ceramic boat at 525 °C for 5 h in open-air conditions (both ends of the tube furnace open), yielding a tannish powder and held at 150 °C prior to removal from the tube furnace to ensure a water-free surface. To yield more consistent oxidations, a low-cost 4 in. fan was positioned at the entrance to force fresh air through the tube furnace environment. Post oxidation yields were typically 60–65%. The NDs were then placed in a glass scintillation vial and inserted into a drying oven (∼140 °C) to prevent water adsorption prior to bromination chemistry. Dry (water-free) ND–OH samples were stored in a drying oven until needed for further chemistry or spectroscopic characterization.

#### Glassware Preparation, Solvent Preparation

To minimize water contamination, all synthetic glassware, including 100 and 1000 mL single-neck round-bottom flasks, Pasteur pipettes, scintillation vials, stir bars, and glass adapters were dried in a 140 °C drying oven to remove water 24 h prior to the start of the bromination. Micropipettes or micropipette tips containing any rubber or plastic pieces were dried at 40 °C in a vacuum oven for 24 h. Anhydrous solvents were poured over molecular sieves that were activated at 200 °C in vacuo prior to the reactions into 1000 mL round-bottom flasks. The work was performed in an N_2_ gas-filled Inert Technologies HE-Purelab 4-port glovebox (Amesbury, MA).

#### ND-Br Synthesis/Purification

Reactions of dry ND–OH with thionyl bromide (SOBr_2_) were set up inside a N_2_ glovebox (GB) and then transferred to a Schlenk line for 24 h. To increase bromination rates, pyridine was used as a mild catalyst in anhydrous DCM. Pyridine-catalyzed reactions were performed by charging a round-bottom flask with 40 mg of ND–OH, 0.516 mL of SOBr_2_ [0.6 M], 5.467 mL of DCM, and 0.591 mL of pyridine in the GB. Reaction flasks were sealed with a glass adapter with vacuum grease, and metal keck clips were attached; the reaction was then removed from the inert atmosphere glovebox and placed in a cup horn sonicator (Fisher Scientific FB505). Cup horn sonication was done at 75% power for 5 min (5 s on/off cycles) and immediately vortexed for 1 min to fully disperse the mixture. The samples were then attached to a purged Schlenk line and stirred vigorously at 500 rpm with a N_2_ flow rate of approximately 1 mL/min (1 bubble per second) and allowed to react for 24 h at 25 °C. At the 6 h mark, reactions would be removed from the Schlenk line, cup horn sonicated for 2 min, and then returned to the Schlenk line for the remainder of the reaction. Three purge and fill cycles were used to remove oxygen and water from the connecting tubing of the Schlenk line to the reaction vessel. After 24 h, the samples were removed from the Schlenk line, sonicated for 5 min using a bath sonicator (Branson 3800), vortexed, and introduced into the glovebox. Because of the instability of the ND-Br constructs, only a bath sonicator was used afterward during purification washes. This was done to prevent debromination and allow for deagglomeration to occur.

Pyridine does produce a side product of pyridinium perbromide salt that was gelatinous and reddish-black in tone and was removed via purification. Typically, three purification cycles with DCM, one with DMSO, and a final wash with DCM concluded the sample workup. DMSO solubilized the salt during the purification cycle and was typically complete after one cycle. Note: If residual dark material was observed, an additional DMSO workup was performed (see S1 in Supporting Information (SI)). Purification of ND-Br solutions was all air-free and done by transferring samples to a 50 mL polypropylene centrifuge (Beckman Coulter# 357003) tube and centrifuging at 21,000 rpm (50,000 rcf) for 30 min at 10 °C. The ND-Br construct was reintroduced into the GB, the supernatant was discarded, and the ND-Br pellet was collected. 15–20 mL of anhydrous DCM or DMSO was added to the ND-Br pellet, bath sonicated, vortexed, and centrifuged again. The purification cycles were completed 4–5 times, during the final wash, the supernatant would be optically clear and colorless. ND-Br samples were stored as solid powders or dispersed in 1 mg/mL in DCM for various experiments. Long-term storage of ND-Br was in a 4 °C refrigerator as a powder or in solution in inert conditions. ND-Br samples were typically reacted with amines within 1–2 days after bromination.

#### ND-NH_3_, ND-DETA, ND-PEI, and ND-MA Preparation

Reaction conditions are summarized in Table 1 and are described fully here. In an inert atmosphere glovebox, a dry 100 mL single-neck round-bottom flask was charged with 10 mL of ND-Br (1 mg/mL) in DCM, 750 μL of ammonia in THF [0.4 M], and a stir bar. In a similar fashion, 38.6 μL, 64.8 μL, or 76.4 mg of polyethylenimine (PEI) (*M_w_* ∼ 800 g/mol), diethylenetriamine (DETA) or melamine (MA) were added in individual reactions, respectively. The flasks were sealed with a septum in the glovebox, lightly bath sonicated for 1–2 min, and secured on a stirplate. Reactions were stirred at 500 rpm, periodically bath sonicated, and completed in 24 h. Workup of ND-NH_3_, ND-PEI, ND-DETA, and ND-MA was performed with 10 mL of anhydrous DCM, for a total of three washes each at 50,000 rcf for 30 min in open-air conditions, and a final wash with 10 mL of 18 MΩ water. The samples were then stored at 1 mg/mL in water.

**Table 1:** Synthesis Conditions for ND Reactions Including the 4 Nucleophile Volumes and Final Concentrations in 10 mL of Anhydrous DCM

nucleophile	sample name	ND-Br mass (mg)	DCM volume (mL)	nucleophile volume	nucleophile concentration
NH_3_/THF	ND-NH_3_	10	10	750 μL	27.9 mM
PEI	ND-PEI	10	10	38.6 μL	5 mM (800 g/mol)
DETA	ND-DETA	10	10	64.8 μL	60 mM
MA	ND-MA	10	10	76.4 mg	60.6 mM

#### Open-Air DRIFTS Using Harrick Praying Mantis

Diffuse reflection infrared Fourier transform spectroscopy (DRIFTS) measurements were performed using a Harrick Praying Mantis DRIFTS attachment (DRK-3), a high-temperature reaction chamber (Harrick #HVC-DRM-5), and a Thermo Fisher FTIR (6700) equipped with an MCT/A detector. OMNIC software was used for instrument control. DRIFTS measurements were performed with 128 scans at a resolution of 2 cm^–1^ and background scans of near or equal signal intensity. KBr powder was stored in an oven at 120 °C for 24 h prior to being used for DRIFTS measurements. Using a mortar and pestle, 80 mg of KBr was ground into a fine powder and introduced into the cup, and the excess KBr was then leveled with a sample preparation tool, leaving a flat and gouge-free KBr surface for background collection. The saved background data were then applied to subsequent data collection. To collect sample data, 3–4 mg of ND–OH or aminated ND samples were added to 80–90 mg of KBr and mixed thoroughly via mortar and pestle. Sample DRIFTS data were collected in percent reflectance mode with the representative background scan.

#### Sample Deposition on Gold-Coated Silicon Wafers for XAS and XPS

Samples were prepared on LGA Thin Films (Santa Clara, CA) gold-coated silicon wafers that were diced into 10 mm × 10 mm pieces. The wafers were cleaned three times by bath sonication in acetone, isopropanol, and last in 18 MΩ water. After sonication, N_2_ gas was used for drying and removing residual water. Next, a piranha treatment of the wafers was performed in a crystallizing dish with 80 mL of piranha solution (1:3 H_2_O_2_/H_2_SO_4_). Postpiranha samples were rinsed with milli-Q water using Teflon tweezers to minimize damage to the gold coating. Sample deposition was performed with two 100 μL cycles of a 1 mg/mL ND-amine solution with the hot plate set at 40 °C to increase water evaporation rates. The wafers were covered with a crystallizing dish to prevent contaminants from reaching the ND surfaces. After drying, they were sent to EAG Laboratories in coin cases for XPS analysis or transferred to the Stanford Synchrotron Radiation Lightsource mounted on an Al sample bar.

#### Laboratory XPS Measurements

A Thermo Scientific K-α Surface Analysis XPS instrument at the Molecular Foundry and EAG Technologies to probe for C 1s, N 1s, and O 1s photoemission. K-α Plus XPS has a combined low-energy electron and ion flood source and is utilized to suppress charging during all data collection. The XPS X-ray source and detector were an Al Kα microfocused monochromator equipped with a 180° double hemispherical analyzer and a 128-channel detector. The low-resolution and high-resolution pass energies were 200.0 and 50.0 eV, respectively. The low-resolution and high-resolution energy step sizes were 1.0 and 0.1 eV, respectively. For high-resolution scans, 50 scans of Br and N were taken, and 10 scans for O and C were taken to ensure a good signal-to-noise ratio, with a dwell time of 50 ms. The electron acceptance angle was 55°, survey scans were performed over a binding-energy range from 0 to 1350 eV, with a pass energy of 200 eV, 3 scans were summed, and a dwell time of 10 ms was used.

#### Synchrotron XAS and RIXS Data Collection

X-ray absorption (XAS) measurements were performed at beamlines 8–2 and 10–1 at the Stanford Synchrotron Radiation Lightsource, SLAC National Accelerator Laboratory, using a spot size of <1 mm^2^. All samples were handled in open-air conditions and mounted to an Al sample bar with conductive carbon tape (#16073-4 Ted Pella, Inc. Redding, CA). The samples were transported to the beamline in a sealed polypropylene jar, and a magnetic mounting piece was attached to the sample bar. Samples were introduced into the analysis chamber after the transfer chamber reached 1 × 10^–7^ Torr. The analysis chamber pressure was typically 5 × 10^–9^ Torr during the measurement.

At beamlines 8–2 and 10–1, C, N, and O K-edge XAS were measured in total electron yield (TEY) mode using 42 × 42, 40 × 40, and 30 × 30 μm slits, respectively. TEY mode probes a depth of a few nanometers. After the optics were focused, the reference absorption intensity of the incoming X-ray beam was measured using a sample of gold-coated mesh and used to correct for beam instability. XAS data were collected at an incident electric field vector of 54.7°. For spectral analysis, the data were treated with a linear pre-edge background subtraction from a region before the absorption edge of C, N, and O at 260–280, 370–380, and 510–530 eV, respectively. Postedge normalization was also performed in the continuum region at 340 eV for C, 420 eV for N, and 580 eV for O, using a batch processing macro in Igor Pro. Energy calibration of the incident X-ray was performed during grating changes with a Ni slab (Ni L3 absorption) and a 1-point fitting procedure. Energy calibration for the C K-edge was further refined using the signal from the diamond core-hole exciton, which was determined to be 289.0 eV as described elsewhere.^25^

Resonant inelastic X-ray scattering or RIXS measurements were performed using the superconducting transition edge sensor (TES) X-ray detector as described elsewhere by Lee and Titus.^26,27^ The TES collects background-free X-rays without a diffraction grating with good energy resolution (approximately 1.5 eV). A RIXS measurement is performed by sweeping across the excitation photon energies controlled at the beamline and collecting a time-tagged X-ray pattern across the TES detector. The TES allows all photons to be collected simultaneously across all detector elements in the array. Analysis of the RIXS data produced by the TES detector is covered thoroughly by Lee et al.

## Results and Discussion

### Mechanism of Bromination and Amination Chemistry

Alkyl bromide termination on HPHT nanodiamond surfaces has been demonstrated to be metastable, while enabling catalyst-free formation of diamond–amine bonds at room temperature.^23^ Bromination of ND–OH surfaces using the strong brominating agent SOBr_2_ proceeds through nucleophilic attack of surface tertiary alcohols on the sulfur center, forming a bromosulfite ether intermediate and liberating bromide ions. Pyridine catalyzes the reaction through sequential deprotonation of the bromosulfite ether and nucleophilic attack on sulfur, producing SO_2_ as a leaving group and generating a carbocation-like surface intermediate that is subsequently attacked by bromides to form ND-Br. Density functional theory (DFT) calculations confirmed that surface-bound bromine is unstable, with a maximum coverage of ∼50%, and that bromine desorption readily regenerates the carbocation intermediate.^24,28^ Because of steric hindrance, high surface energy, and reactivity with water, ND-Br samples must be handled under inert conditions and may undergo debromination within 1–2 weeks even in a glovebox. Despite this instability, the facile bromide desorption makes the surface highly reactive toward nucleophiles, allowing for efficient C–N bond formation. As illustrated in Figure 1, the addition of polyethylenimine (PEI), a branched ∼800 g/mol polydentate amine (∼2 nm in size), quenches the carbocation surface state and forms covalent carbon–nitrogen bonds through nucleophilic attack by primary and secondary amines. The carbocation intermediate is sufficiently long-lived to enable nearly complete conversion of brominated sites in anhydrous dichloromethane at 25 °C. At ∼50% bromine coverage on diamond (111) surfaces (∼18 atoms nm^–2^), approximately nine reactive surface sites are available with ∼500 pm spacing, which is compatible with multidentate attachment of PEI or smaller amines such as DETA at ∼250 to 500 pm intervals.^29–31^

**Figure 1:**
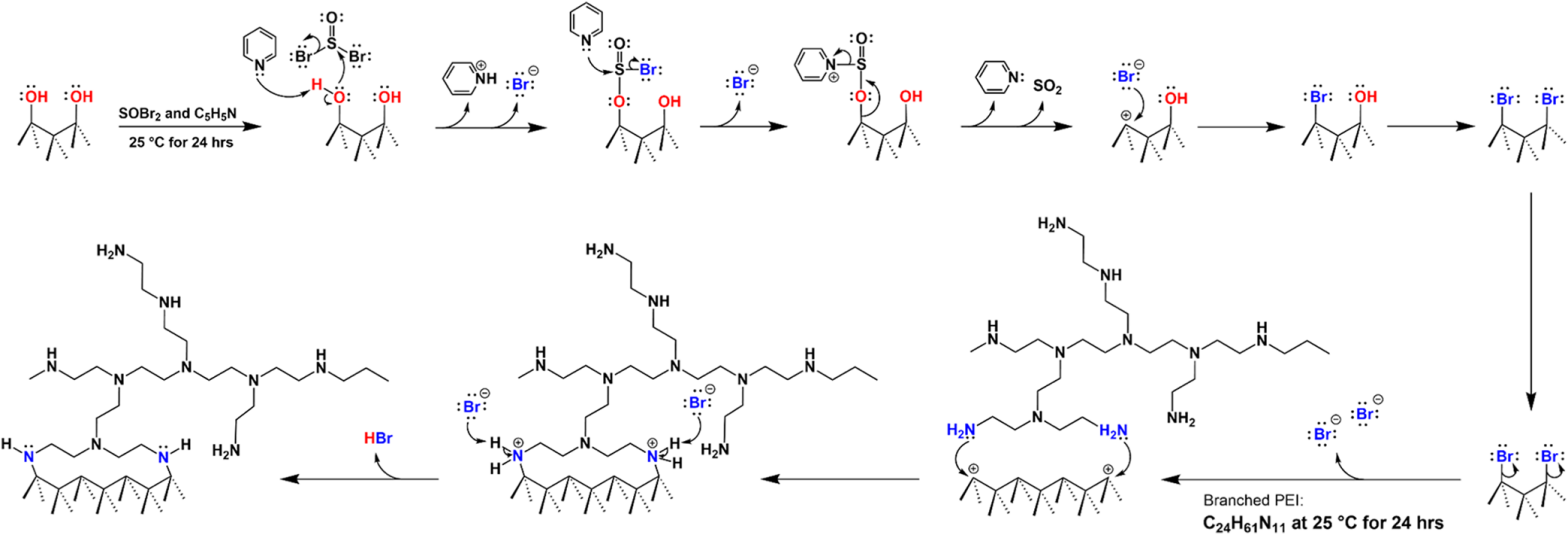
Schematic of bromination and amination on an oxidized HPHT ND surface. The branched PEI molecule performs a nucleophilic attack on carbocations generated by the dissociation of alkyl-bromide in anhydrous DCM at 25 °C over a 24 h period to form ND-PEI with direct amine bonds to the diamond surface.

### Summary of Size, Morphology, and Nucleophilicity of Amines

The molecules selected range in morphology (branched, cyclic, and linear), size (∼0.25 to 2.5 nm), and nucleophilicity. PEI contains primary, secondary, and tertiary amines, with the primary amines boasting the most nucleophilicity.^32^ PEI is used in applications ranging from antimicrobial coatings on nanoparticles to brain imaging studies.^33–35^ DETA is an alkyl amine containing 2 primary amines and 1 secondary amine with a length of only 760 pm and is a tridentate ligand in metal–ligand complexes.^36,37^ MA is unique as a 1,3,5-triazine derivative with 3 substituted amines and is considered a good nucleophile due to π-conjugation of the triazine ring and stabilization of lone pair electrons on nitrogen for enhanced electron localization.^38^ Melamine is used industrially for flooring, as a flame retardant and in the cleaning product “Magic Erasers,” it was also used infamously in a baby formula scandal to increase protein concentration.^39^ When ordering their nucleophilicity based on sterics and stabilization of lone pairs, we rationalize that nucleophilicity increases from DETA to PEI to MA. This is the first study of PEI, DETA, and MA reactivity on diamond and provides a range of properties that are potentially valuable in biolabeling and sensing applications.

### DRIFTS of ND-NH_3_, ND-PEI, and ND-DETA

DRIFTS is a convenient technique to quickly screen samples and confirm the conversion of ND–OH into ND-amine-terminated constructs. Our starting material ND–OH is produced by aerobic oxidation and shows a strong (C–O)_ν_ peak at 1105 cm^–1^, a minor (C=O)_ν_ peak at 1780 cm^–1^, a (O–H)_δ_ peak at 1640 cm^–1^, and a (O–H)_ν_ band from 3000 to 3500 cm^–1^ that are assigned to alcohols, carboxylic acids, adsorbed water and alcohols/adsorbed water, respectively (Figure 1).^40^ Decreases in intensity of the (C–O)_ν_ peak, changes in adsorbed water, and emergence of new features in comparison to ND–OH are a qualitative measure of amine chemistry success. Spectra are given in Kubelka–Munk (KM) units and are linear with concentration if quantification is needed. First, we examined ND-NH_3_ and observed the elimination of the 1105 cm^–1^ peak of alcohols and the emergence of a (C–N)_ν_ peak at 1025 cm^–1^ due to the addition of amines.^23^ An increase in the O–H stretching band and bending mode at 1640 cm^–1^ was also observed, indicating that water adsorption may be increasing on the diamond surface. Next, we observed the ND-DETA spectrum had a drastic reduction in (O–H)_ν_ band absorption due to decreased hydrophilicity of the diamond surface in concert with sharp molecular features of (C–H)_ν_ at 2900–3000 cm^–1^, and (C–H)_δ_, (N–C)_ν_ and deformation modes in the fingerprint region from 650 to 1500 cm^–1^ and was consistent with standardized samples. Two ND-DETA features were not consistent, including the (N–H)_ν_ mode at ∼3300 cm^–1^, which was broad and reduced in intensity, while a ∼900 cm^–1^ feature was not observed, potentially due to the coupling of the primary amines to the diamond surface. In contrast, ND-PEI showed increased stretching mode absorption from 2500 to 3700 cm^–1^ due to (C–H)_ν_, (N–H)_ν_, and water adsorption features.^41^ Similar to ND-DETA, the (N–H)_ν_ mode at ∼3300 cm^–1^ in ND-PEI was reduced by ∼50% in comparison to standard PEI samples and was also reported with detonation NDs coated in PEI.^42^ These observations do suggest that primary amine-related stretching and bending modes are suppressed when they interact with the ND surface.

#### DFT of Melamine Clusters Shows H-Bonding-Dependent Spectra

The covalent addition of melamine (MA) to the surface is unique, and vibrational modes activated by hydrogen bonding (H-bonding) provide insights into the intermolecular forces at play. Based on DFT, isolated melamine molecules without intermolecular H-bonding do not have the experimentally observed features (MA-1). In Figure 2a, the ND-MA vibrational regions are highlighted in pink and then represented in greater detail in Figure 2b,c in comparison with DFT-based vibrational spectra. The calculated FTIR spectra by Yuan et al. use 1-molecule (MA-1), 4-molecule (MA-4), and 32-molecule (MA-32) clusters with 0, 2, and 30 H-bonds, respectively.^43^ Our ND-MA spectra are similar to the bulk FTIR spectra taken of high-purity melamine and indicate a close-packed arrangement of MA on the diamond surface for a number of key reasons. Importantly, the vibrational modes of ND-MA would not be observed unless H-bonding was active, with H-bonding interactions scaling as 1/*r* ^3^. H-bonding occurs with interaction lengths of *r* = 0.15–0.3 nm, supporting the close spacing of melamine molecules within that range.^44^ Additionally, a previous spectroscopic study by Mircescu also highlights the difference between single molecule FTIR and a cluster approach, which buttresses this interpretation.^45^

**Figure 2:**
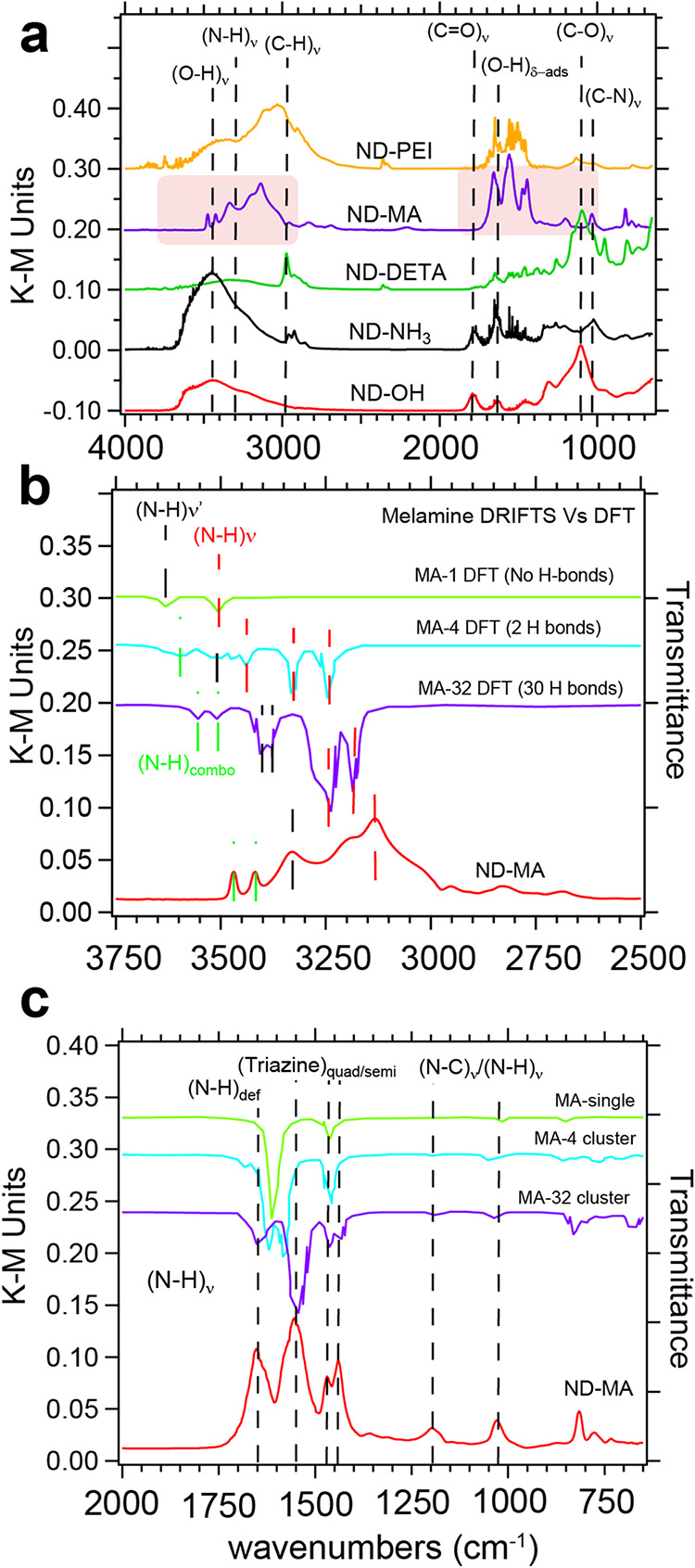
DRIFTS and transmittance spectra for ND-NH_3_, ND-PEI, ND-DETA, ND-MA, and DFT calculated melamine vibrational modes. Kubelka–Munk units are used for the analysis of amine functionalization and quantification. After amination of ND-Br, reductions in C–OH modes and increases in C–N modes were observed along with sharp molecular vibrations also being observed (a). We compare the H-bonding dependent modes of melamine based on DFT with our experimental spectra of ND-MA (b, c). Changes in hydrophilicity were also apparent based on the molecular decoration of the ND sample. Reprinted (Adapted or Reprinted in part) with permission from X. Yuan, K. Luo, K. Zhang, J. He, Y. Zhao, and D. Yu, J. Phys. Chem. A 2016, 120, 7427–7433.

#### ND-MA Modes Are H-Bonding Dependent at Short Length Scales

First, let us examine the band region from 2500 to 3750 cm^–1^ in Figure 2b where we compare 4 spectra: MA-1, MA-4, and MA-32 in conjunction with ND-MA. MA-1 without hydrogen bonding has higher vibrational energies than all data sets and progressively decreases as cluster size and H-bonding increase. To guide the vibrational progression, we use black dashed lines for asymmetric N–H modes, red for symmetric N–H modes, and green for combination modes. MA-1 without H-bonding has only 2 vibrational modes at 3621 and 3494 cm^–1^ due to asymmetric (N–H)_ν‑asym_ and symmetric (N–H)_ν‑sym_ modes, respectively. The (N–H)_ν‑asym_ (black lines) of MA-1 → ND-MA decrease from 3621 cm^–1^ to 3518 cm^–1^ to 3393/3370 cm^–1^ to 3331 cm^–1^ and are due to the *M*_υ as_^1^ + *M*_υ as_^2^ combination modes, where *M* refers to N–H modes. In a similar fashion, (N–H)_ν‑sym_ (red lines) also decreases from 3494 cm^–1^ to 3426/3318/3233 cm^–1^ to 3221/3170 cm^–1^ to 3129 cm^–1^ based on a pure *M*_υs_^1^ vibration with increasing H-bonding. One would expect a decrease in vibrational energy with increased H-bonding based on distance, angle, and quantity.^46^ Lastly, the green dashed lines depict the combination modes with more complex molecular motion than classic symmetric and asymmetric stretches. Described as *M*_υ as_^3^ + *M*_υ as_^4^ and *M*_υ as_^5^ + *M*_υ as_^6^, these modes decrease from 3494 cm^–1^ to 3420 cm^–1^ in MA-32 and ND-MA; and 3595 cm^–1^ to 3555 cm^–1^ to 3468 cm^–1^ in MA-4, MA-32 and ND-MA, respectively. To picture the *M*_υ as_^3^ + *M*_υ as_^4^ vibrational eigen vectors, one depicts this as a simultaneous small elongation of one N–H bond in combination with a major compression of H–N on the same primary amine. While MA-32 does not depict the experimental ND-MA exactly, the H-bonding trends are well established and provide us a view into the melamine-diamond interface. There is also no strong evidence of water adsorption based on stretching modes around 3400–3700 cm^–1^, which suggests a decrease in the hydrophilicity of the ND-MA samples.

As the picture of closely interacting MA molecules on the diamond surface emerges, the fingerprint region provides triazine ring modes, C–N stretches, and deformation modes that are less sensitive to H-bonding (Figure 2c). The calculated spectra of the models from MA-1 to MA-32 fall within the region of the experimental ND-MA results immediately. Starting at high energy, the features in this region are the (N–H)_def_ mode at 1650 cm^–1^ and the 1–3–5 triazine stretching modes at 1548, 1469, and 1438 cm^–1^ (Figure 2c). To illustrate the combinatorial modes, the 1548 cm^–1^ peak is a quadrant stretching mode that is the sum of *M*_β_ + *T*_β_^1,3^ + *T*_ν_^2^ + *P*_ν_, wherein *M*, *T*, and *P* are the N–H bond, out-of-ring C–N bond and in-ring C–N bond, respectively.^43^ At lower energy, we have good agreement between the MA-32 and ND-MA features for (C–N)_ν_ and (N–H)_ν_ at 1195 and 1026 cm^–1^, respectively. Low-energy modes around 800 cm^–1^ are understood to be the (C–N)_δ_ and (N–H)_δ_ bending modes and again are in good agreement between the calculations and experimental data sets.

Based on DRIFTS and the DFT conclusions discussed above, the distances between MA molecules across the diamond surface are in the range of 0.15–0.3 nm, and, due to steric constraints, they are arranged normal to the surface with ∼5 to 6 MA molecules per nm^2^. The edge-on stacking is consistent with covalent bond formation by the triazine lone pair or amines after bromide desorption. Temperature-dependent desorption with mass spectroscopy could be used to determine the bond type and strength in a later study.

#### XPS Provides Semi-Quantitative Insights for ND-Amine Bond Formation

XPS spectra revealed that the amine reactivity increased from NH_3_ to PEI to DETA to MA and is understood based on nucleophilicity and sterics. XPS provides surface-sensitive and element-specific spectra with a probe depth of ∼1 to 2 nm using a commercial instrument; here, we use XPS to quantify the contribution of elements using survey scans and also confirm the bonding environment of amine molecules covalently bound to the diamond surface (see Figures 1 and 3). Quantification of C 1s, N 1s, and O 1s signals with CasaXPS can be extracted using eq 2 and Table S1 in the SI.^47^ Table 2 summarizes the % atomic concentrations for carbon, nitrogen, and oxygen. The starting material, ND–OH, has carbon and oxygen % concentrations of ∼86 and 14%, respectively, with an estimated error of 10%; after amination chemistry, the contribution of nitrogen varies from 1.4 to 8.65% based on the nucleophile used. The simplest conversion of ND-Br to ND-NH_3_ shows C 1s, N 1s, and O 1s values of 82.3, 1.4, and 16.3%, respectively. The increase in O 1s can be understood by the increased water adsorption of the ND-NH_3_ surface and is supported by increased O–H band absorption about 3000 cm^–1^ (see Figure 2a). ND-PEI and ND-DETA contain N 1s atomic concentrations of 2.8 and 1.6%, respectively, indicating that fewer PEI-800 molecules were bound to the surface than expected. Every PEI-800 has on average 18 nitrogen molecules, while DETA has only 3. We speculate that the branched PEI-800 did not access the ND surface as efficiently due to steric hindrance and is unexpected, considering PEI-coated detonation ND studies are highly successful due to electrostatics.^42^ These ND-PEI results highlight a major difference between the covalent attachment of a branched polymer versus a simple coating due to charge–charge interactions.

**Figure 3:**
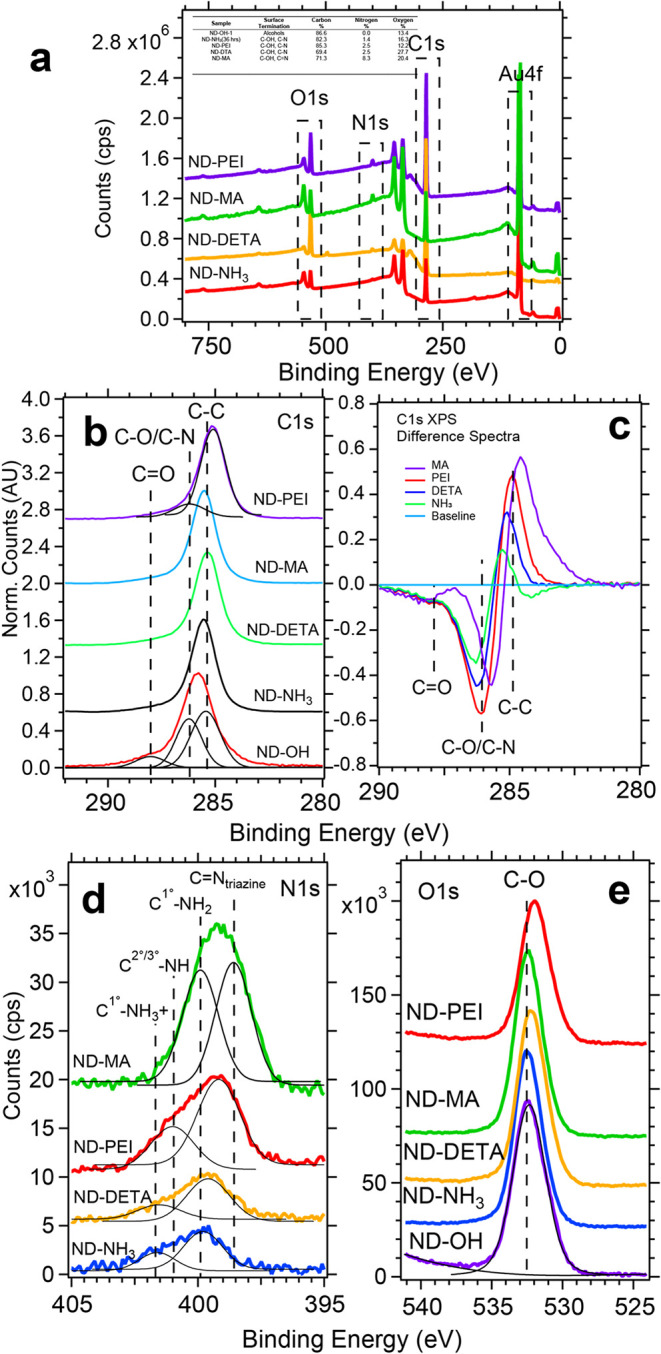
Survey and high-resolution XPS scans show the quantitative contribution of nitrogen to the surface and change in diamond surface chemistry. Survey XPS showed nitrogen ranged from 1 to 8% based on the nucleophile and melamine (MA) (a). High-resolution C 1s spectra (b) and difference spectra of the C 1s showed a subtle shift to lower binding energy due to reduced C–O bond formation and changes in the surface charge and surface dipole moment (c). N 1s XPS spectra revealed contributions from primary, secondary, and tertiary carbon–nitrogen bonds in conjunction with protonated species (d). O 1s XPS was consistent with peak positions in the 532.0–532.5 eV range, indicative of C–O bonds (e).

**Table 2:** Quantitative Summary of XPS Survey Scans Using VAMAS File Types for % Atomic Concentration Analysis (10% Error Assumed for All Values)

sample	surface termination	carbon %	nitrogen % (N/molecule)	oxygen %
ND-OH-1	alcohols	86.6	0.0	13.4
ND-NH_3_ (36 h)	C–OH, C–N	82.3	1.4 (1)	16.3
ND-PEI	C–OH, C–N	84.8	2.8 (18)	12.4
ND-DETA	C–OH, C–N	82.3	1.6 (3)	16.1
ND-MA	C–OH, C–N, C=N	71.3	8.3 (6)	20.4

Surprisingly, melamine showed the largest nitrogen content at 8.3%, while one would expect the 800 g/mol PEI to dominate, because of its multidentate nature and increased degrees of freedom. Melamine being a triazine derivative has increased nucleophilicity due to enhanced electron density to the lone pair electrons within the ring.^48,49^ The 1,3,5-triazine ring reactivity and the hydrogen-bonding signatures from DRIFTS further support our vertical alignment theory. Notably, the atomic % of O 1s was slightly increased to 20.4%, and we speculate that a reduction of C 1s photoemitted electrons from diamond played a role and was not due to increased hydrophilicity or water adsorption. DRIFTS showed “dry” ND-MA samples, with H-bonding occurring between MA molecules alone (see Figure 2a,b DRIFTS spectra).

#### High-Resolution C 1s Shifts Follow Nucleophilicity of Amines

High-resolution XPS spectra of the C 1s and O 1s regions indicate overlapping contributions from oxidized diamond and amine functionalization, while N 1s spectra confirm nucleophile incorporation. The C 1s spectra (Figure 3b) show modest changes following conversion from ND–OH to ND–PEI and remain dominated by sp^3^ C–C bonding at ∼285.4 eV. Notably, carbon atoms bonded to oxygen- and nitrogen-containing functional groups (alcohol, amine, imine, and carboxyl species) appear between 286.2 and 288.0 eV. Because these features substantially overlap, quantitative deconvolution was not performed. Alternatively, difference spectra relative to ND–OH (Figure 3c; background-subtracted and normalized) showed a decreased photoemission intensity above 285.6 eV, consistent with reduced C–O contributions, and a maximum shift to lower binding energy (284.6 eV) for ND–MA. These results indicate the formation of a heterogeneous surface containing both C–O and C–N bonds, with concurrent protonation–deprotonation equilibria involving alcohol and amine groups altering both charge identity and quantity.^50^

The binding-energy shift is attributed to a reduction in the average surface dipole moment and mixed charge character associated with alkoxide and ammonium surface species. A systematic trend (NH_3_ → DETA → PEI → MA) is observed, consistent with increasing nucleophilicity associated with secondary amine functionality. All samples were dispersed in 18 MΩ water at pH 7.5–8.0; amine p*K*_a_ values must therefore be considered when evaluating protonation of the surface moieties. Prior studies of hydrogen- and oxygen-terminated diamond surfaces have reported subtle C–C binding-energy shifts by XPS, although the origin of these shifts remains unresolved, even for single-crystal diamond substrates.^50,51^

#### N 1s XPS Identifies 4 Species of Bonding Structure

Next, we examined the N 1s spectra with conformation of the amines, triazine rings, and evidence of protonation generating NH^+^ moieties. Each molecule has varying p*K*_a_ values as mild bases with 1-aminoadamantane (molecular analogue of ND-NH_3_), DETA, PEI, and MA of 10.1, 9.58, 8–9.9, and 5.0, respectively.^52^ For the sake of clarity, C^1°^-NH_2_ and C^1°^-NH_3_+ are primary amines in unprotonated and protonated forms; C^2°/3°^-NH and C=N_triazine_ are secondary/tertiary amines and imines within the melamine structure in Figure 3d. ND-NH_3_ and ND-DETA spectra have a similar structure with C^1°^-NH_2_ and C^1°^-NH_3_+ peaks found at ∼399.9 and ∼401.7 eV, respectively, and are consistent with previous amine-terminated diamond samples.^17,23^ The protonated C^1°^-NH_3_+ feature has a significant shift to higher binding energy (∼1.8 eV) and is 31% and 25% of ND-NH_3_ and ND-DETA integrated peak intensities, respectively.

Branched ND-PEI is the most complicated molecule in this study and has primary, secondary, and tertiary amines (see Figure 1). Peak fits of ND-PEI at 399.2 and 401 eV are assigned to the C^1°^-NH_2_ and C^2°/3°^-NH bonding environments, respectively. While no fit was used for a C^1°^-NH_3_+ assignment, it is reasonable that the species is present.^53^ Melamine is unique in having both imines and primary amines present with peaks at 398.5 and 399.8 eV, both in equal intensity. The expected C^1°^-NH_3_+ peak did not arise and is rationalized by the poor solubility of melamine in water (27 mM at 25 °C) and low basicity (p*K*_a_ = 5.0).^54,55^ Overall, the O 1s spectra of all samples in Figure 3e were little changed from sample to sample with the dominant C–O feature found in the range of 532.0–532.5 eV, consistent with some tertiary alcohols remaining on the surface post-amination.

#### XAS Data Shows Strong N 1s Signal and Midgap State Absorption

Valuable information regarding the electronic structure of molecules and solids, including bond order, bond length, and orientation of adsorbates, is determined through XAS, an element-specific spectroscopy technique.^56^ When collecting data in total electron yield (TEY) mode, XAS provides surface and bulk information simultaneously with a probing depth of 5–10 nanometers in the soft X-ray regime. Diamond features observed in all C 1s XAS spectra include the characteristic core-hole exciton and second bandgap at 289.0 and ∼302 eV, respectively (Figure 4a).^25,57^

**Figure 4:**
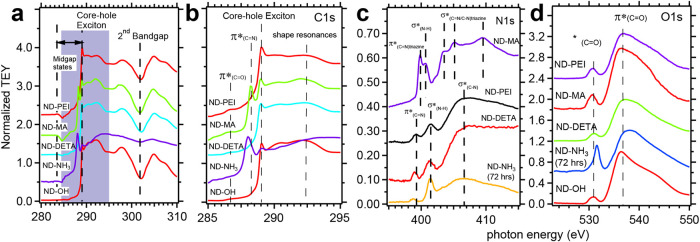
XAS spectra shows bulk and surface information for ND-OH and samples with covalently bound DETA, PEI, MA, and PGA. C 1s XAS shows the diamond electronic structure (i.e. core hole exciton and 2nd absolute bandgap) and midgap states that are highlighted by the arrows from 283.5–289 eV (3a/b).15 New mid gap states are observed with ND-NH_3_ and ND-MA samples along with π*­(C=O) states. N 1s XAS reveals C=N, C–N and N–H π* and σ* of the aminated samples with strong TEY counts (3c). O 1s XAS spectra shows subtle changes in the C=O and C–O bonding environments due to removal of alcohols, water desorption/adsorption and unknown factors (3d).

Unoccupied midgap states within the 5.5 eV diamond bandgap are highlighted by the double-ended arrow and include features below 289 eV. The purple region is then reproduced in Figure 4b to highlight the pre-edge absorption features, including the weak π*_(C=O)_ at 286.5 and 288.1 eV due to carboxylic acids. ND-NH_3_ and ND-MA show strong pre-edge absorption at 288.0 and 288.25 eV, respectively, and are assigned to the π*_(C=N)_ resonance of imines and the triazine ring. While the source of π*_(C=N)_ within ND-MA is clear, the strong feature in ND-NH_3_ (72 h reaction) is poorly understood. One possibility is the insertion of NH_3_ across sp^2^-like Pandey chains similar to hydroamination of alkenes, but the mechanism is beyond the scope of this work.^58^ Another plausible mechanism is due to X-ray beam damage, causing deprotonation and C=N bond formation. Interestingly, the diamond signal of ND-NH_3_ was reduced by ∼75% in comparison to ND–OH and could be a result of a shell structure or high charge density that prevents TEY collection. Previous reduction in diamond TEY signal occurred with SiO_2_ shells on ND cores and reactions of ND-Br with propargylamine.^23,59^

Starting with the control ND-NH_3_ spectra, we observe N 1s peaks at 398.6 and 401.4 eV assigned to the π*_(C=N)_ and σ*_(N–H)_ bonding environments due to X-ray beam damage (i.e., minor deprotonation and imine bond formation) and the amine-terminated surface, respectively. The shape resonance at 406.6 eV is assigned to the σ*_(C–N)_ bond due to the diamond-amine covalency. Next, ND-DETA includes both primary and secondary amines and has similar π*_(C=N)_, σ*_(N–H)_, and σ*_(C–N)_ features at 399, 401.3, and 406.8 eV, respectively. Comparisons to NH_3_ in the gas phase and solvated NH_3_ in water reveal that the N 1s XAS line shapes and peak positions suggest that hydrogen bonding plays a role.^60^ The branched ND-PEI and ND-DETA are almost identical in peak position and intensity and reinforce the similarities in the bonding structure between the molecular and polymeric systems.

In stark contrast, ND-MA has a unique electronic structure that reflects both the triazine ring and the three amines bonded to carbons 2, 4, and 6 of melamine. Five peaks were observed at 399.9, 400.6, 403.6, 405.2, and 409.5 eV and can be segmented into π* and σ* regions. The ultrasharp peak at 399.9 eV is consistent with the lowest unoccupied molecular orbital (LUMO) level of the triazine ring and is referenced as π*_(C=N)triazine_.^61^ The σ*_(N–H)_ peak at 400.6 eV is ∼0.7 eV lower in energy than the nonconjugated systems and reflects the lowering of energy due to intramolecular hydrogen bonding as observed using DRIFTS. Higher energy transitions in the range 403.6–409.5 eV are assigned to the σ*_(C=N/C–N)triazine_ that reflects the 1s → p states of the ring system. The lowest energy transition at 399.9 eV is believed to be excitonic based on DFT calculations and RIXS data that are found in SI Section 4. Based on our results, melamine on diamond would be a model system for a number of fundamental studies: electron donation to the diamond surface and Fermi level shifts, detection of ^14^N or ^15^N NMR sensing with NV centers, and electron transfer studies for photocatalytic investigations.

To round out the surface characterization, O 1s XAS was collected and found to be largely unchanged between aminated samples with π*_(C=O)_ and σ*_(C–O)_ features at ∼530.7 and 536 eV representative of carboxylic acids and the remaining alcohols postchemical modification. The lone anomaly in O 1s XAS was the ND-NH_3_ sample after 72 h of amination chemistry, which showed increased intensity in the region of 530–533 eV. We speculate that this increase could be due to the conversion of an acid bromide to an amide or a combination of π* oxygen-bonding environments. Previous use of NH_3_ in THF as a nucleophile showed little change in the π*_(C=O)_ resonances and indicates that with longer reaction times (i.e., 72 h versus 24 h), nitrogen insertion at the surface increases, simultaneously producing amide-like moieties.

#### Potential Uses in NV Sensing and Photocatalysis

Covalent attachment of PEI, DETA, and MA provides tunability of the dipole moment on diamond surfaces under mild conditions that should enhance electron-spin coherence times (see Chou and Galli) and produce solvated electrons.^14,17^ While plasma-based amination chemistry is quick, milder wet chemistry techniques provide broader molecular control and no surface roughening. To that point, NV center characterization is needed to confirm that amination is fully beneficial. During review, the authors located additional plasma-based amination articles and one wherein the NV center characteristics were enhanced.^62–65^ The change in the Fermi energy of the aminated ND surfaces is needed to determine the electron affinity characteristics of the aminated ND surfaces and their ability to produce solvated electrons as well.

## Conclusions

In this diamond surface chemistry study, we expanded the types of amine molecules capable of covalent bond formation via the ND-Br intermediate. Using linear, branched, and cyclic amines, there is evidence that secondary amines of DETA and PEI, along with the nitrogen lone pairs of the triazine ring of MA, are responsible for the new diamond-nitrogen bonds. Empirically, the reactivity increases from NH_3_ to PEI to DETA to MA and can be rationalized by p*K*_a_, nucleophilicity due to electron donation, and steric considerations. Hydrogen bonding also played a role in increasing hydrophilicity in the case of ND-NH_3_ and ND-PEI solubilized in water, while intermolecular hydrogen bonding generated in ND-MA was evidenced by DRIFTS spectra. ND-NH_3_ and ND-DETA samples showed ammonium groups present due to increased basicity, which was confirmed by N 1s XPS. XAS confirmed both surface and bulk diamond information, and a π*_(C=N)_ state was found at ∼288 to 288.2 eV for the NH_3_- and MA-treated samples that represents an electron acceptor state ∼1 eV below the conduction band minimum of diamond and 0.4 eV below the nitrogen vacancy center’s first excited state. This work expands the chemical pathways for covalent ND surface decoration, and researchers in the field of quantum sensing and photocatalysis may leverage these findings to tune surface dipole moments, charge identity, hydrophilicity/hydrophobicity, and electron affinity.

## Supplementary Material



## Data Availability

Original data sets are available through Mendeley data and can be accessed here: DOI: 10.17632/v2c3fh6bx6.1.

## Notes

Summary of the bromination routes in carbon nanomaterials, Kubelka–Munk transformation procedures, XPS data analysis procedures, additional DRIFTS data relating to pyridinium perbromide on HPHT NDs, and RIXS maps (PDF)
