# Supplementary material for: Magic Diamond: Covalent Bond Formation of Melamine and Other Amines on Nanodiamond Surfaces
Source: ACS Omega. 2026 Apr 17;11(17):25526–36. doi: 10.1021/acsomega.5c13652 (PMC13150610; doi:10.1021/acsomega.5c13652)
Supplement: Supplementary file 1 [file ao5c13652_si_001.pdf]

Supporting Online Material for

Magic Diamond: Covalent Bond Formation of Melamine and other Amines on Nanodiamond Surfaces

Tsz Ching Cheung [1,ξ], Camron X. Stokes[1, ξ], Jorge A. Lopez-Rosas [1,ξ], Grace Olivia Drew [1], Joy Lillian Drew [1], Anoushka Lakshmi[1], Nivita Susendran[1], Muhammed Qasim[1], Cynthia Melendrez[4], Sang-Jun Lee[3], Avery Green[7], Jia Lu[8], Virginia Altoe[6], Dennis Nordlund[3], Kent Irwin[3,5], & Abraham Wolcott[1,2*]

[1] Department of Chemistry
San José State University
1 Washington Square
San José, CA 95192, U.S.A.

[2]Department of Physics
CUNY-The City College of New York
160 Covent Ave.
New York, NY 10031, USA.

[3] Stanford Synchrotron Radiation Lightsource
SLAC National Accelerator Laboratory
2575 Sand Hill Road
Menlo Park, CA 94025

[4] Linac Coherent Light Source
SLAC National Accelerator Laboratory
2575 Sand Hill Road
Menlo Park, CA 94025

[5] Stanford University
Department of Physics
450 Jane Stanford Way
Palo Alto, CA 94025

[6] The Molecular Foundry
Lawrence Berkeley National Laboratory
1 Cyclotron Road
Berkeley, CA U.S.A.

[7]Covalent Metrology
927 Thompson Pl
Sunnyvale, CA 94085, U.S.A.

[8]EAG Laboratories
810 Kifer Rd
Sunnyvale, CA 94086, U.S.A.

* Corresponding authors: A. Wolcott (awolcott@ccny.cuny.edu)
ξ equal contributions

Table of Contents

1. Summary of bromination routes in carbon nanomaterials page S3

2. KM Transformation Procedure and XPS Quantification page S4-5

3. DRIFTS of residual pyridinium perbromide on HPHT NDs page S6

4. RIXS, PFY-XAS and XES spectra page S7-9

5. References page S10

1. Summary of bromination of carbon nanomaterials (non-diamond):

Bromination of other carbon-based nanomaterials such as carbon nanotubes (CNTs), graphene and graphene quantum dots (GQDs) has been more widely used in contrast to nanoscale diamond. Vapor phase bromoalkylation of double-walled carbon nanotubes (DWCNTs) was accomplished by reacting the DWCNTs with Br_2 vapor at room temperature for a week.[1] Bulusheva et al. found that the brominated DWCNTs slowly reduce in weight as temperature increases, and the bonding energy of the bromine changes, highlighting a rearrangement on the CNT surface.[1] Wet chemical bromination of carbon nanodots (CNDs) occurred via 5M hydrobromic acid (HBr) treatment, placing them in an impinger bottle for 6 hours.[2] Plasma-enhanced bromination of graphene and graphite by Friedrich et al. was performed in an elastomer-sealed, tube-shaped glass reactor.[3] A plasma frequency of 13.56 MHz was used, creating an exothermal reaction enthalpy to promote C-Br bond formation which primarily occurs through electrophilic addition to aromatic double bonds. From the single C-Br bonds, successful conversion from C-Br to hydroxyl groups and amino groups was demonstrated.[3,4] That route is similar to the work done by Melendrez et al. in which metastable brominated nanodiamonds were generated to serve as a labile moiety for amine bond formation.[1]

2. DRIFTS and XPS Data Analysis Procedures:

DRIFTS and KM transformation. Kubelka–Munk (KM) transformations were performed individually with linear background corrections in Igor Pro software. Linear backgrounds were generated based on the averaged values of percent reflectance (raw data) in the DRIFTS regions of 2000–2200 cm^{-1} and 3800–4000 cm^{-1}. This slope value was then applied to a y-intercept function (y=mx+b) and applied to spectra for normalized reflectance units (R) and then transformed using the Kubelka–Munk equation to generate KM units that are proportional to concentration:

$$KM\ Units = \frac{(1-R)^2}{2R} \quad (1)$$

As a reference, infrared values were cross referenced to *infrared and Raman characteristic group frequencies: tables and charts* by Socrates, HPHT ND studies and the NIST Webbook values.[5-9]

XPS Quantification procedure.

XPS analysis was performed using Igor Pro software and the CasaXPS software package, and standard background subtraction and fitting protocols were followed on survey and high-resolution scans. For example, a linear background subtraction was performed on N1s high-resolution scans in Igor Pro, and then, peaks were fit to a Voigt line shape with a mixed Gaussian and Lorentzian contribution. Peak widths were typically held to a full width at half-maximum (FWHM) of 1.5–2.0 eV as appropriate for the spectral features. Quantitative analysis of survey scans to determine the atomic percentage of individual elements was performed using CasaXPS software, and Tougaard backgrounds were applied with relative sensitive factor (RSF) values being applied for each element. The RSF values for C, N, O and Br were 1.0, 1.8 and 2.93, respectively. The percentage atomic concentration (X_A) values were calculated using the following equation:

$$X_A = \frac{(I_A E^{\alpha})/(R_A T(E))}{\Sigma(I_i E^{\alpha})/(R_i T(E))} \quad (2)$$

where X is the atomic percentage of element A, R_i is the RSF for the relative intensity I_i and T(E) is the transmission function of the instrument for intensity I_i at kinetic energy (E).[10] The alpha term in the exponent of kinetic energy E is used to adjust for analyzer specifications. The VAMAS (Versailles Project on Advanced Materials and Standards) file collected by the K-Alpha instrument allowed for all needed transmission function information to be applied for quantification purposes, and the VAMAS file type was ISO 14976 compliant.

Table S1: Inelastic mean free paths of C1s, N1s, O1s and Br3d electrons based on Seah and sensitivity factors based on transmission functions in VAMAS files.

Element	Edge	IMPF (nm)	Sensitivity factors
**Carbon**	1s	1.87	1.0
**Nitrogen**	1s	1.78	1.8
**Oxygen**	1s	1.67	2.93

3. **DRIFTS of Pyridinium perbromide contamination.**

Contamination of pyridinium perbromide (PPBr) was identified with DRIFTS and showed sharp vibrational peaks and a broad stretching mode band from 2500-3500 cm^{-1}. The PPBr material left on the HPHT ND surface looks very similar to the standards of PPBr (PPBr FTIR) with a series of finger print region stretching modes and a near identical band of the $(C\text{-}H)_\nu$ and $(N\text{-}H)_\nu$ modes. After bromination the ND-Br spectrum is dominated by broader features of surface bound moieties including the

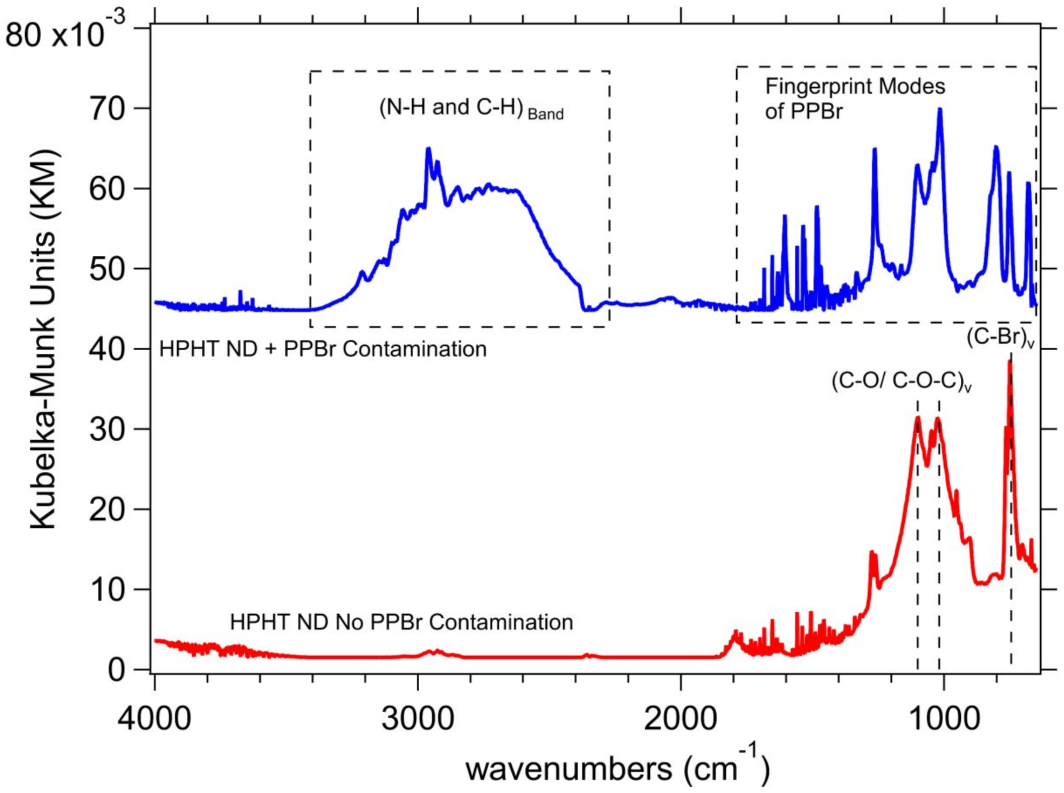

Figure S1. DRIFTS spectrum of HPHT NDs with residual pyridinium perbromide (PPBr) and without residual contamination. Note the massive band of stretching modes from almost 2500-3200 cm-1 consistent with standardized PPBr samples. The $(C\text{-}Br)_\nu$ mode along with XPS and other spectroscopies confirmed the tertiary bromide termination.

4. Resonant Inelastic X-Ray Data and Analysis:

RIXS maps of Melamine on Diamond. RIXS maps provide occupied and unoccupied density of states (DOS) of a material or molecular system and is powerful in connecting specific absorption and emission features.[13] In this first RIXS study of melamine several unique features are observed while ND-DETA is also probed. RIXS maps are generated by varying the incoming X-ray radiation and collecting the X-ray emission with the TES detector. Analysis of the RIXS map is performed by vertical (red boxes) or horizontal (white boxes) integration windows that yield the partial fluorescence yield X-ray absorption spectroscopy (PFY-XAS) and X-ray emission spectroscopy (XES) data sets, respectively. Figure S2 is a monochromator corrected RIXS map and the red boxes outline the integrated count region as a function of excitation energy (vertical integration) with a Rayleigh scattering feature in the lower right corner. Once done, the integrated region produces a PFY-XAS spectrum as seen in Fig. S2B/C with regions from 383-391 eV and 391.6-399 eV. The electronic bandgap of melamine can also be estimated by locating the lowest/highest energy feature in the PFY-XAS and XES spectra with an estimate of 5.5 eV. Next, we can deconvolve the contributions of the 399.9 eV and 401 eV peaks originally observed in the XAS spectrum by integrating different emission windows (ie. red boxed regions). The resonantly excited state at 399.9 eV is coupled to emissive states from 392.8-394.9 eV, while the 401 eV peak is coupled to a diffuse emissive DOS from 386-390.5 eV. Above 402 eV the σ* states dominate the PFY-XAS spectra with XES features spanning a wider range of 392.5-396.5 eV and 383-391.5 eV, respectively. Unfortunately, we do not have the counts and spectral resolution to observe vibronic features of melamine, yet there is enough energy resolution in the ionization edge of ~400 eV to connect specific features with DFT calculated orbitals.

Lowest π* transition of ND-melamine is excitonic. Based on previous DFT calculations using 6-311++G(d,p) basis sets for the highest occupied molecular orbitals (HOMO) and lowest unoccupied molecular orbitals (LUMO) energy levels, the intense resonant feature at 399.9 eV is likely excitonic and matches well with a 6.45 eV bandgap of melamine as reported by Prabhaharan.[14] The LUMO states responsible for the excitonic transition are localized on the triazine ring p_z orbitals and is likely the $1s \rightarrow \pi^*(C_2\text{-}N_3)$ transition. In that same study, natural bond order calculations that describe intramolecular electron donor-acceptor orbitals revealed that a large stabilization energy ($E^{(2)}$) of 52 kJ/mol was found with $\pi(N_1\text{-}C_6) \rightarrow \pi^*(C_2\text{-}N_3)$ orbital overlap

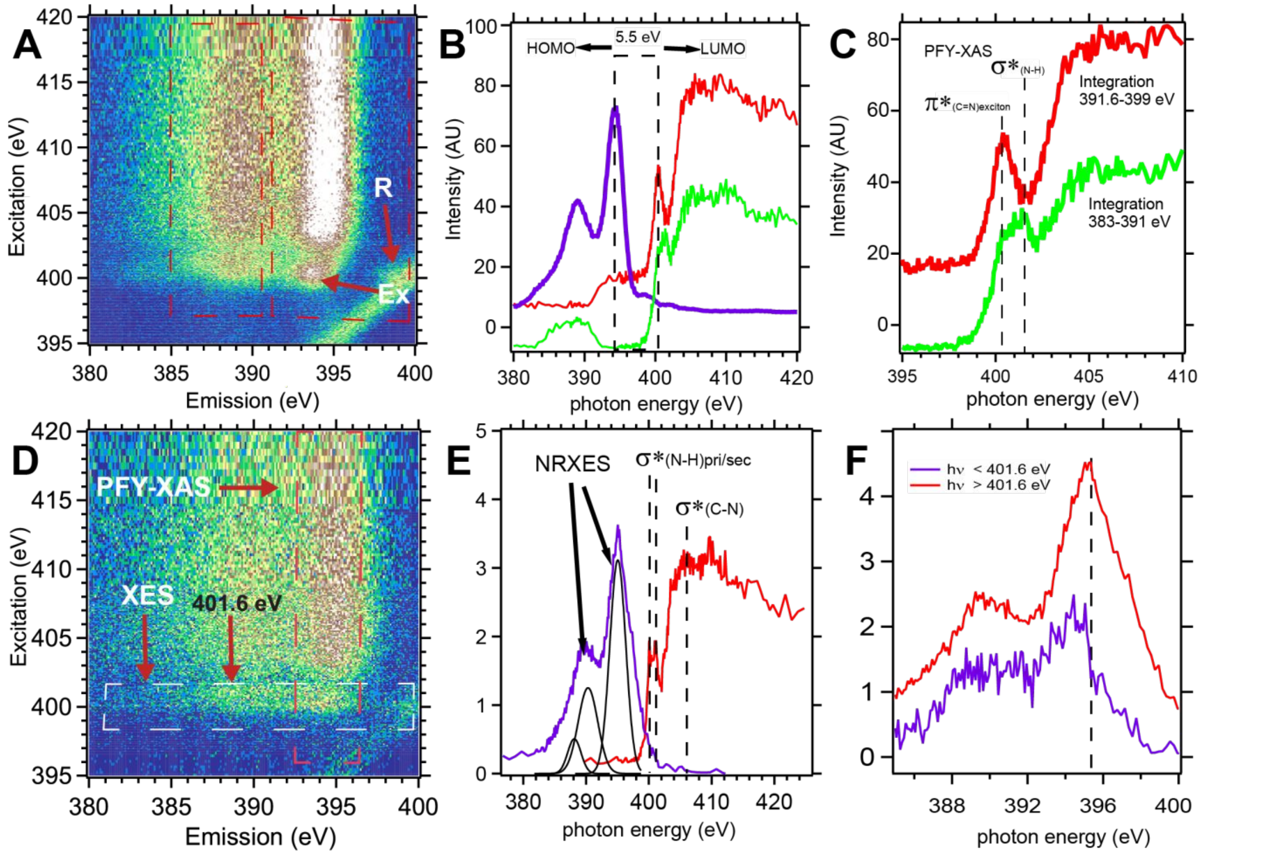

Figure S2. RIXS maps of ND-MA and ND-DETA show the full occupied and unoccupied density of states in a single data set. Integration of counts vertically produces a PFY-XAS spectrum while horizontal integration produces a XES spectrum. Conformation of an excitonic state in ND-MA at 399.9 eV of ND-MA and contributions to the $\sigma^*(N\text{-}H)_{pri/sec}$ states of ND-DETA were observed with RIXS.

and reinforces the origins of the 399.9 eV peak. With this evidence, we propose the sharp C1s peak at 288.2 eV (see fig. 4B) as the $\pi^*(C{=}N)_{triazine}$ transition of ND-MA (labelled Ex). Surprisingly, the 288.2 eV peak competes in intensity to that of the core-hole exciton of diamond. This unoccupied state is approximately 0.8-1 eV below the conduction band minimum of diamond and near equal in energy to the vibrationally relaxed 1st excited state of the NV center. Investigating the effect of melamine as an electron acceptor on the surface of diamond and its impact on NV center electron-spin properties would be a natural avenue to explore. Another characteristic to explore is the electron affinity (EF) of the diamond-melamine and whether positive or negative EF is present.

DETA bound to the diamond surface contains RIXS features less intense than that of ND-MA and does not show the $\pi^*(C{=}N)$ transition around 398 eV found during XAS measurements

(Figure S2). PFY-XAS spectra of ND-DETA does reveal two distinct peaks at 400.1 eV and 401.2 eV that are assigned to the σ*(N-H) resonances for both primary and secondary amines. A shape resonance at ~406 eV is then assigned to the σ*(C-N) states (Figure S2E). When examining the XES spectra we selected X-ray emission with monochromator values above or below 401.6 eV (Figure S2F). The purpose was to deconvolve contributions in the HOMO states and recognize subtle shits in XES similar to the primary and secondary amine peaks in the PFY-XAS. Photon energies above 401.6 eV did contribute to greater XES intensity and peak locations of 395.1 eV and 389.5 eV (red trace). Decreasing incoming X-rays to below 401.6 eV resulted in a decrease in overall XES intensity and a shift of the high energy peak by 0.5 eV to 394.6 eV. These results suggest that access to these HOMO states are largely dominated by deexcitation from C-N bonds and that N-H states contribute less to the XES signal. Further studies with higher energy resolution can reveal the vibronic contributions of the DETA bound to the diamond surface.

References:

1 Bulusheva, L. G. *et al.* Bromination of Double-Walled Carbon Nanotubes. *Chemistry of Materials* **24**, 2708-2715 (2012). https://doi.org:10.1021/cm3006309

2 Knoblauch, R., Harvey, A. & Geddes, C. D. Metal-Enhanced Photosensitization of Singlet Oxygen (ME1O2) from Brominated Carbon Nanodots on Silver Nanoparticle Substrates. *Plasmonics* **16**, 1765-1772 (2021). https://doi.org:10.1007/s11468-021-01438-1

3 Friedrich, J. F. *et al.* Plasma-chemical bromination of graphitic materials and its use for subsequent functionalization and grafting of organic molecules. *Carbon* **48**, 3884-3894 (2010). https://doi.org:https://doi.org/10.1016/j.carbon.2010.06.054

4 Friedrich, J. F., Hidde, G., Lippitz, A. & Unger, W. E. S. Plasma Bromination of Graphene for Covalent Bonding of Organic Molecules. *Plasma Chemistry and Plasma Processing* **34**, 621-645 (2014). https://doi.org:10.1007/s11090-013-9509-x

5 Socrates, G. *Infrared and Raman characteristic group frequencies:tables and charts.* 3rd edn, (John Wiley and Sons, 2001).

6 Osswald, S., Yushin, G., Mochalin, V., Kucheyev, S. O. & Gogotsi, Y. Control of sp(2)/sp(3) carbon ratio and surface chemistry of nanodiamond powders by selective oxidation in air. *Journal of the American Chemical Society* **128**, 11635-11642 (2006). https://doi.org:10.1021/ja063303n

7 Krueger, A. & Lang, D. Functionality is Key: Recent Progress in the Surface Modification of Nanodiamond. *Advanced Functional Materials* **22**, 890-906 (2012). https://doi.org:10.1002/adfm.201102670

8 Krueger, A., Stegk, J., Liang, Y., Lu, L. & Jarre, G. Biotinylated nanodiamond: Simple and efficient functionalization of detonation diamond. *Langmuir* **24**, 4200-4204 (2008). https://doi.org:10.1021/la703482v

9 Girard, H. A. *et al.* Surface properties of hydrogenated nanodiamonds: a chemical investigation. *Physical Chemistry Chemical Physics* **13**, 11517-11523 (2011). https://doi.org:10.1039/c1cp20424f

10 CasaXPS. *CasaXPS User's Manual*. 42-54 (2001).

11 Yuan, X. *et al.* Combinatorial Vibration-Mode Assignment for the FTIR Spectrum of Crystalline Melamine: A Strategic Approach toward Theoretical IR Vibrational Calculations of Triazine-Based Compounds. *The Journal of Physical Chemistry A* **120**, 7427-7433 (2016). https://doi.org:10.1021/acs.jpca.6b06015

12 Schmitz, C. H., Ikonomov, J. & Sokolowski, M. Two commensurate hydrogen-bonded monolayer structures of melamine on Ag(111). *Surface Science* **605**, 1-6 (2011). https://doi.org:https://doi.org/10.1016/j.susc.2010.09.006

13 Ament, L. J. P., van Veenendaal, M., Devereaux, T. P., Hill, J. P. & van den Brink, J. Resonant inelastic x-ray scattering studies of elementary excitations. *Reviews of Modern Physics* **83**, 705-767 (2011). https://doi.org:10.1103/RevModPhys.83.705

14 Prabhaharan, M., Prabakaran, A. R., Gunasekaran, S. & Srinivasan, S. Molecular structure and vibrational spectroscopic investigation of melamine using DFT theory calculations. *Spectrochimica Acta Part A: Molecular and Biomolecular Spectroscopy* **123**, 392-401 (2014). https://doi.org:https://doi.org/10.1016/j.saa.2013.12.056
